# Preying on seals pushes killer whales from Norway above pollution effects thresholds

**DOI:** 10.1038/s41598-020-68659-y

**Published:** 2020-07-17

**Authors:** Clare Andvik, Eve Jourdain, Anders Ruus, Jan L. Lyche, Richard Karoliussen, Katrine Borgå

**Affiliations:** 1 Department of Biosciences, University of Oslo, 0316 Oslo, Norway; 2 Norwegian Orca Survey, Daniel Olaisensvei 6, 8480 Andenes, Norway; 3 Norwegian Institute of Water Research, Gaustadalléen 21, 0349 Oslo, Norway; 4 Department of Food Safety and Infection Biology, Norwegian University of Life Sciences, Campus Adamstuen, P.O. Box 5003, 1432 Ås, Norway

**Keywords:** Marine biology, Stable isotope analysis, Food webs, Environmental impact, Environmental monitoring

## Abstract

Killer whales (*Orcinus orca*) are at risk from high levels of biomagnifying pollutants, such as polychlorinated biphenyls (PCBs) and mercury (Hg). Previous toxicological risk assessments for the Norwegian killer whale population have assumed fish as the primary prey source, and assessed the population as below established effect thresholds. However, some individuals have recently been identified to also feed on seals. This study is the first to quantify levels of pollutants in seal-eating killer whales from northern Norway, and to measure Hg levels in the skin of killer whales worldwide. We found higher levels of all pollutants in seal-eating than fish-eating killer whales, including the emerging brominated flame retardants pentabromoethylbenzene (PBEB), pentabromotoluene (PBT) and hexabromobenzene (HBB). Sum polychlorinated biphenyls (ΣPCBs) in the blubber of seal-eaters (*n* = 7, geometric mean = 46 µg/g l.w.) were four times higher than fish-eaters (*n* = 24, geometric mean = 11 µg/g l.w.), which pushed all seal-eating individuals above multiple thresholds for health effects. Total Hg levels in skin of seal-eaters (*n* = 10, arithmetic mean = 3.7 µg/g d.w.) were twice as high as in fish-eaters (*n* = 28, arithmetic mean = 1.8 µg/g d.w.). Our results indicate that by feeding on higher trophic prey, the Norwegian killer whale population is at higher risk of health effects from pollution than previously assumed.

## Introduction

Pollutants, such as polychlorinated biphenyls (PCBs) and mercury (Hg), have the potential to cause harmful effects in wildlife due to their persistent, bioaccumulative and toxic properties^1,2^. PCBs are a type of organohalogen contaminant (OHC): anthropogenically produced pollutants that include a range of pesticides and industrial products. Hg is a naturally occurring element, however the concentrations measured in wildlife have increased by 3–4 orders of magnitude over the last 150 years due to anthropogenic activities^3^.

The killer whale (*Orcinus orca*) is a species particularly vulnerable to biomagnifying pollutants due to its position as an apex predator, long life span, and thick blubber layer in which lipophilic OHCs can accumulate^4^. High recorded tissue levels of PCBs and Hg led to the killer whale being named the most exposed species by the Arctic Monitoring and Assessment Programme (AMAP)^5^, and over 50% of killer whale populations worldwide have been predicted to collapse within the next 100 years due to PCB exposure^6^. The levels of pollutants in a killer whale population, and thus its risk of health effects, are higher in populations feeding at high trophic levels due to increased exposure from biomagnification in prey^6,7^. For example, in the northeast Pacific, marine-mammal eating killer whale populations have higher levels of PCBs than fish-eating populations from the same geographic area^8,9^. Variations in Hg levels according to dietary preference have not yet been recorded in killer whales, although higher Hg levels would be expected in groups feeding on marine mammal prey, as Hg also biomagnifies in food webs^10,11^.

Killer whales frequenting the coast of Norway have long been assumed to feed primarily on Atlantic herring (*Clupea harengus*)^12,13^. However, field observations and stable isotope dietary descriptors have recently confirmed that some individuals switch from fish to marine mammal prey, such as seals, on a long term and multi-seasonal basis^14,15^. Toxicological risk assessments of the Norwegian killer whale population have been based on the PCB levels of nine, fish-eating killer whales sampled from one area of northern Norway in 2002^5,6,16^. Hg levels have not been measured to date. With recent knowledge of the varied dietary preferences in the Norwegian killer whale population, along with changing environmental exposure, a quantification of the PCB and Hg levels of both fish and seal-eating killer whales is necessary for more accurate risk assessments.

The aim of this study was to quantify and compare the PCB levels in blubber, and total Hg levels in skin, of seal-eating and fish-eating killer whales in northern Norway. The assignment of whales to dietary groups was based on stable isotope analysis and observational data presented in a previous study^14^. Our results are relevant in conducting a more accurate risk assessment of contaminant-mediated health effects in this population, accounting for heterogeneity in foraging habits.

## Results and discussion

### PCB levels

**Figure 1:**
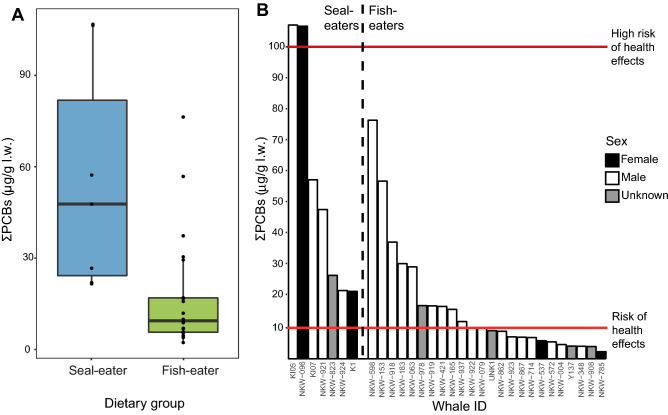
ΣPCB levels (µg/g lipid weight) in blubber of seal-eating (*n* = 7) and fish-eating (*n* = 24) killer whales from Norway. (**a**) Levels in dietary groups. Horizontal lines represent the median, whiskers represent the lower (first) and upper (fourth) quartiles, dots represent individual whales, points outside the whiskers and box are outliers. (**b**) Levels in each individual whale, ordered on the x-axis in decreasing levels of ΣPCBs within dietary groups. The red horizontal lines (10 μg/g l.w. and 100 μg/g l.w) are, respectively, the thresholds for a risk, and high risk, of health effects^7^.

We found higher sum of PCBs (ΣPCBs) levels in seal-eating killer whales than fish-eaters (Welch’s t-test, *t* = 4.47, *p* = 0.0006; Fig. 1a, Table 1). The geometric mean ΣPCBs for adult male seal-eaters (*n* = 4; 46 µg/g l.w) was almost double the levels previously recorded in adult male fish-eating killer whales from Norway in 2002 (*n* = 8; 27 µg/g l.w)^16^. These lower levels from 2002 have been used to conclude that killer whales from Norway are at less risk from PCB-mediated health effects than other Arctic killer whale populations^7^. We found higher median ΣPCBs in adult male seal-eating killer whales from our study (51 µg/g l.w), which were comparable to levels in an adult male killer whale harvested from Greenland with seal remains in the stomach (65 µg/g l.w), suggesting equitable risk^17^.

**Table 1:** Summary of lipid-normalised concentrations (µg/g) of organohalogen contaminants in sampled killer whales (*n* = 31).

OHC group	Seal-eaters			Fish-eaters		
	All^f^ (*n* = 7)	Adult male (*n* = 4)	Adult female (*n* = 2)	All^f^ (*n* = 24)	Adult male (*n* = 17)	Adult female (*n* = 2)
**ΣPCBs**^a^						
μ ± SD	56.0 ± 37.8	58.9 ± 35.9	64.6	16.7 ± 18.3	20.7 ± 20.4	4.07
Geometric mean	45.7	50.7	48.4	10.9	14.2	3.66
Median (range)	48.2 (21.8–108)	53.0 (22.0–108)	64.6 (21.8–107)	9.52 (2.3–77.1)	12.0 (4.03–77.1)	4.07 (2.3–5.85)
**ΣDDTs**^b^						
μ ± SD	28.3 ± 21.1	30.9 ± 16.7	32.9	15.3 ± 21.4	19.3 ± 20.4	2.49
Geometric mean	21.4	27.7	19.9	8.59	11.4	2.20
Median (range)	26.8 (6.67–59.2)	27.5 (14.4–54.2)	32.9 (6.67–59.2)	7.79 (1.33–100)	8.96 (3.04–100)	2.49 (1.33–3.65)
**ΣCHLs**^c^						
μ ± SD	9.78 ± 5.59	10.3 ± 3.75	11.6	4.35 ± 5.01	5.31 ± 5.65	1.17
Geometric mean	8.41	9.69	9.13	2.98	3.80	0.952
Median (range)	9.46 (4.06–18.9)	10.6 (5.54–14.4)	11.7 (4.41–18.9)	2.67 (0.489–23.4)	3.06 (1.52–23.4)	1.17 (0.489–1.85)
**HCB**						
μ ± SD	0.523 ± 0.467	0.389 ± 0.309	0.893	0.205 ± 0.0903	0.229 ± 0.0934	0.105
Geometric mean	0.401	0.324	0.650	0.18	0.216	0.0973
Median (range)	0.290 (0.220–0.146)	0.242 (0.220–0.852)	0.873 (0.290–1.45)	0.178 (0.0649–0.519)	0.194 (0.126–0.519)	0.105 (0.0649–0.146)
**β-HCH**						
μ ± SD	0.116 ± 0.090	0.0974 ± 0.0692	0.179	0.0470 ± 0.0369	0.0546 ± 0.0935	0.0186
Geometric mean	0.0929	0.0827	0.145	0.0378	0.0456	0.0146
Median (range)	0.0739 (0.0426–0.284)	0.074 (0.0426–0.199)	0.179 (0.0739–0.284)	0.0311 (< LOD–0.193)	0.0513 (< LOD–0.193)	0.0186 (< LOD–0.030)
**Mirex**						
μ ± SD	0.295 ± 0.148	0.256 ± 0.179	0.400	0.0700 ± 0.0777	0.0842 ± 0.0879	0.0384
Geometric mean	0.224	0.166	0.398	0.0425	0.0522	0.383
Median (range)	0.347 (< LOD–0.446)	0.283 (< LOD–0.437)	0.400 (0.354–0.446)	0.0449 (< LOD–0.350)	0.0478 (< LOD–0.304)	0.0384 (< LOD–0.0417)
**ΣPBDEs**^d^						
μ ± SD	1.57 ± 0.73	1.61 ± 0.39	1.67	0.554 ± 0.415	0.491 ± 0.444	0.218
Geometric mean	1.41	1.58	1.22	0.447	0.553	0.180
Median (range)	1.38 (0.529–2.81)	1.51 (1.27–2.13)	1.67 (0.529–2.81)	0.466 (0.0960–1.79)	0.491 (0.258–1.79)	0.218 (0.0960–0.339)
**HBB**						
μ ± SD	0.009 ± 0.003	0.009 ± 0.004	0.010	0.003 ± 0.003	0.003 ± 0.004	0.002
Geometric mean	0.009	0.009	0.010	0.002	0.00	0.001
Median (range)	0.009 (0.006–0.014)	0.009 (0.006–0.014)	0.010 (0.010–0.010)	(0.0006–0.014)	0.002 (0.0006–0.014)	0.002 (0.001–0.002)
**4′-OH-BDE49**						
μ ± SD	0.008 ± 0.006	0.007 ± 0.004	0.010	0.003 ± 0.003	0.004 ± 0.004	0.0006
Geometric mean	0.006	0.006	0.007	0.001	0.002	0.0005
Median (range)	0.006 (0.002–0.018)	0.007 (0.002–0.011)	0.010 (0.003–0.018)	0.002 (< LOD–0.014)	0.003 (< LOD–0.014)	0.0006 (< LOD–0.0009)
**ΣOHCs**^e^						
μ ± SD	96.6 ± 65.0	103 ± 56.2	112	37.3 ± 44.6	46.3 ± 50.2	8.11
Geometric mean	78.6	91.0	80.6	23.5	30.7	7.17
Median (range)	90.1 (34.1–191)	93.9 (43.6–178)	112 (34.1–191)	20.5 (4.31–202)	24.5 (9.10–202)	8.11 (4.31–11.9)

*LOD* Limit of detection.

^a^Sum of PCB-28, -66, -74, -87, -99, -101, -105, -110, -114, -118, -128, -137, -138, -141, -149, -151, -153, -156-, 157, 170, -180, -183, -187, -189, 194, -196, -206, -209.

^b^Sum of p,p′-DDT, p,p′-DDD and p,p′-DDE.

^c^Sum of oxychlordane, *trans-*chlordane, *cis-*chlordane, *trans-*nonachlor and *cis-*nonachlor.

^d^Sum of BDE-28, -47, -99, -100, -153, -154.

^e^Sum of all organohalogen contaminants detected in more than 65% of whale samples.

^f^Including whales of unknown sex, subadults, and of unknown age: summary statistics for these whales can be found in Supplementary Table S3.

PCBs are associated with immune suppression in marine mammals^18,19^, and all the seal-eating killer whales from our study had ΣPCBs in blubber exceeding the 10 µg/g l.w threshold for a risk of immune and hormone system health effects, determined from laboratory rat studies^7^, in contrast to only 46% of the fish-eating whales (Fig. 1b). Two of the seal-eating killer whales were even above the higher threshold for an elevated risk of health effects (100 µg/g l.w.). All seal-eating killer whales in our study (compared to 54% of fish-eaters) exceeded an additional threshold established using immune system endpoints in feeding experiments on harbour seals (*Phoca vitulina),* of 9 µg/g l.w.^20,21^. The higher levels of PCBs in seal-eaters thus indicate potential weaknesses in the ability to fight diseases, which can have implications for survival and population growth^22,23^.

PCBs also affect reproduction and offspring mortality in wildlife^23,24^. In a recent study, the effects of PCBs on population growth was assessed via influences on calf survival and immune suppression by placing killer whale populations into exposure groups based on the ΣPCB levels in adult female killer whales^6^. The Norwegian killer whale population was placed in the lowest exposure group, 1, based on an estimated ΣPCB value in adult females of 10 µg/g l.w., extrapolated down from measured levels in adult males^6,16^. Populations in exposure group 1 were modelled to double in size within the next 100 years, if model assumptions are accepted. In our study, however, the two adult seal-eating female killer whales had ΣPCB values of 22 µg/g l.w. and 107 µg/g l.w., which would place them in exposure groups 2 and 5, respectively. Populations in exposure group 2 are estimated to have modest population growth, albeit at a reduced rate, whereas populations in group 5 are estimated to be at high risk of population collapse within the next 100 years^6^. The geometric mean ΣPCB levels of all the seal-eating killer whales (46 µg/g l.w.), furthermore, exceeded the ΣPCB threshold of 41 µg/g l.w., at which reproductive impairment with occlusions in the uterus and increased glucocorticoid production in the adrenals was observed in ringed seals (*Pusa hispida*)^21,24^. Killer whales from Norway are observed with viable offspring, and have an estimated calving rate of 0.197^25^. However, this is based on killer whales studied on herring overwintering grounds, which are thus likely fish-eaters. Although we have a small sample size for adult female killer whales (just two individuals in each dietary group, with a large range), the higher levels in seal-eating killer whales may result in increased calf mortality. The difference in the geometric mean ΣPCB levels in female seal-eaters (48 µg/g l.w.) and female fish-eaters (3.6 µg/g l.w.) is translatable to approximately 45% and 80% of calves surviving, respectively^23^. Median ΣPCB levels in female seal-eaters (67 µg/g l.w.) are furthermore comparable to the median ΣPCB levels in female, tuna-eating, killer whales from the Strait of Gibraltar (51 µg/g l.w.), which has one of the lowest reproductive rates for killer whales globally^21^. Lower reproductive rates in the seal-eating killer whales are relevant for the wider ecosystem, as seal-eating killer whales occupy a differentiated ecological niche than the fish-eaters, that could be lost with localised extinctions^14^.

### Mercury

**Figure 2:**
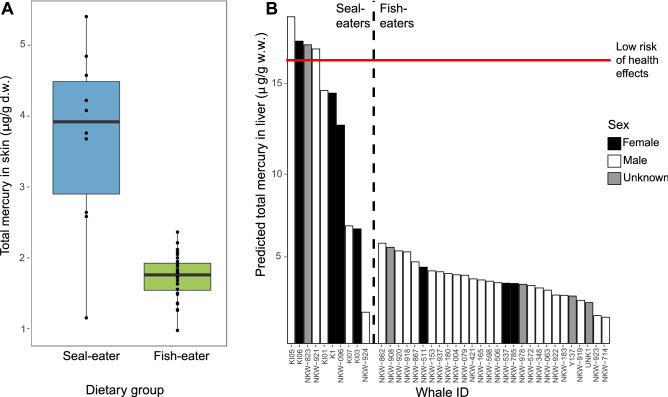
Total mercury levels in fish-eating (*n* = 28) and seal-eating (*n* = 10) killer whales from Norway. (**a**) Levels in skin (μg/g d.w.). Horizontal lines represent the median, whiskers represent the lower (first) and upper (fourth) quartiles, dots represent individual whales, points outside the whiskers and box are outliers. (**b**) Levels in liver (μg/g w.w.), estimated from skin using equation $\ln \left( {Hg_{liver} } \right) = 1.6124 \times \ln \left( {Hg_{skin} } \right) + 2.0346$ based on bottlenose dolphins (*Tursiops truncatus*)^58^, and ordered on the x-axis in decreasing levels of total mercury within dietary groups. The red horizontal line (16 μg/g w.w.) is the threshold for a low risk of health effects in marine mammals, and ranges to 64 μg/g w.w. Levels below the threshold of 16 μg/g w.w. are classified as no risk of mercury-mediated health effects in marine mammals^7^.

Seal-eating killer whales from our study had higher total Hg levels in skin than fish-eaters (arithmetic mean seal-eaters = 3.7 µg/g d.w. and fish-eaters = 1.8 µg/g d.w.; Welch’s t-test, *t* = 4.89, *p* = 0.0008; Fig. 2a, Table 2). The estimated hepatic total Hg levels for both seal-eaters (arithmetic mean: 13 µg/g w.w.) and fish-eaters (arithmetic mean: 3.6 µg/g w.w.) were below the threshold for liver abnormalities in bottlenose dolphins (*Tursiops truncatus*) (61 μg/g w.w.)^26^, and below the threshold for a low risk of health effects in marine mammals (16 µg/g w.w.)^7^, based on toxic hepatitis, uremia and renal failure observed in harp seals (*Pagophilus groenlandicus*)^27^. However, four of the seal-eaters (40%) were above the 16 µg/g w.w. threshold, compared to none of the fish-eaters, still indicating a higher risk of health effects for the seal-eating group (Fig. 2b). This is the first indication of mercury levels in killer whales from Norway to date. It is also the first study worldwide to measure Hg levels in the skin of killer whales, thus allowing for repeatable and minimally invasive biomonitoring. Whilst levels appear low, the extrapolation from liver to skin in our study has uncertainties, and the skin levels are comparable to at-risk populations of bottlenose dolphins from the English Channel^28^ and Mediterranean^29^. There was a positive correlation between the total Hg levels measured in the skin and the total OHC levels measured in the blubber of the whales (Spearman’s rank correlation, *S* = 2,572, *p* = 0.006, rho = 0.48).

**Table 2:** Summary of total mercury levels in sampled killer whales (*n* = 38).

Total mercury levels	Seal-eaters			Fish-eaters		
	All^a^ (*n* = 10)	Adult male (*n* = 5)	Adult female (*n* = 4)	All^a^ (*n* = 28)	Adult male (*n* = 20)	Adult female (*n* = 3)
**Measured in skin (µg/g d.w.)**						
μ ± SD	3.73 ± 1.26	3.52 ± 1.66	3.97 ± 0.95	1.76 ± 0.32	1.78 ± 0.27	3.19 ± 1.49
Geometric mean	3.47	3.11	3.87	1.73	1.76	2.98
Median (range)	3.95 (1.19–5.44)	3.71 (1.19–5.44)	4.18 (2.62–4.88)	1.79 (1.01–2.40)	1.83 (1.29–2.25)	2.62 (2.08–4.88)
**Estimated in liver (µg/g w.w.)**^b^						
μ ± SD	12.5 ± 5.62	11.6 ± 7.1	12.5 ± 4.47	3.57 ± 1.05	3.68 ± 0.987	3.70 ± 0.531
Geometric mean	10.5	8.77	11.8	3.41	3.54	3.68
Median (range)	14.2 (1.78–18.5)	14.3 (1.78–18.5)	13.2 (6.48–17.1)	3.47 (1.49–5.65)	3.61 (1.57–5.65)	3.411 (3.38–4.31)

^a^Including whales of unknown sex, subadults, and of unknown age: summary statistics for these whales found in Supplementary Table S4.

^b^Estimated using equation $\ln \left( {Hg_{liver} } \right) = 1.6124 \times \ln \left( {Hg_{skin} } \right) + 2.0346$ based on bottlenose dolphins (*Tursiops truncatus*)^58^.

### Other organohalogen contaminants

**Figure 3:**
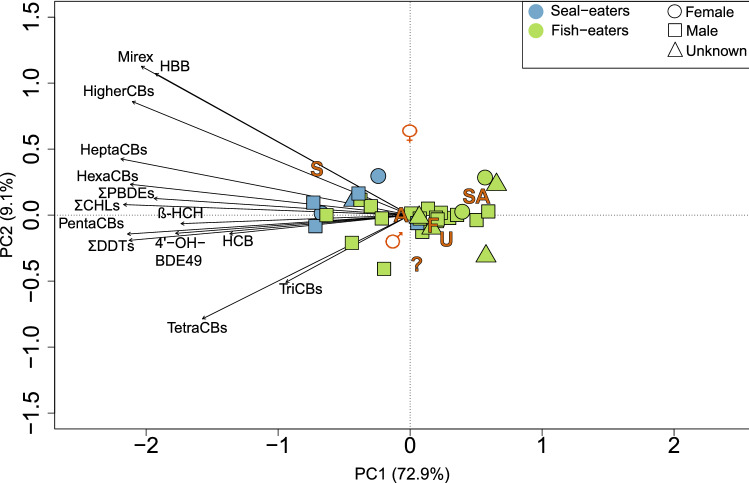
Principal component analysis (PCA) biplot based on organohalogen contaminant levels in blubber of killer whales from Norway. Response loadings are represented as black arrows, and passive explanatory variables are added as orange symbols representing the centroids of the variable (age, sex, dietary group). “S” refers to the centroid for seal-eaters (*n* = 7) and “F” for fish-eaters (*n* = 24). The centroid for males (*n* = 22), females (*n* = 4) and unknown sex (*n* = 5) are represented as ♂, ♀ and U, respectively. “A” refers to the centroid for the adult whales (*n* = 25), “SA” for subadults (*n* = 3), and “?” if the age is unknown (*n* = 3). The percentage of the total variation explained by PC1 and PC2 are given in brackets on each axis.

Levels of other organohalogen contaminants (OHCs), including the pesticide dichlorodiphenyltrichloroethane (DDT) and the industrial chemicals polybrominated diphenyl ethers (PBDEs) were also all higher in the seal-eating killer whales than the fish-eaters (RDA, *F*_1,28_ = 10.53, *p* = 0.001; Fig. 3). Threshold levels for possible toxic effects in marine mammals have not been established for these pollutants. However, many have been linked to oestrogenic, reproductive and endocrine effects in wildlife^30,31^. The Stockholm Convention on Persistent Organic Pollutants has banned many of these pollutants^32^, and whilst downward trends have been observed in the environment^33^, levels are still high and the geometric mean ΣPCB (14 µg/g l.w.), ΣDDT (11 µg/g l.w.) and ΣPBDE (0.55 µg/g l.w.) levels in adult male fish-eating killer whales sampled for this study were comparable to geometric mean levels in blubber of male killer whales sampled in Norway in 2002 (ΣPCB: 27 µg/g l.w., p,p′-DDE: 11 µg/g l.w., ΣPBDE: 0.48 µg/g l.w.)^16^. We also screened for four emerging brominated contaminants in the tissues of both seal and fish-eating sampled killer whales. Whilst levels of 3-dibromopropyl-2,4,6-tribromophenyl ether (DPTE) were below the limit of detection in all whales, pentabromoethylbenzene (PBEB) was found in one whale (a seal-eater), pentabromotoluene (PBT) was found in 58% of the sampled whales (86% of the seal-eaters and 50% of the fish-eaters) and hexabromobenzene (HBB) was found in all individuals. HBB, included in the PCA, was correlated to the other organohalogen contaminants (Fig. 3). The presence of these emerging contaminants in top predators is a concern, as production is unregulated and the effects on marine mammals unknown.

ΣPCBs and ΣDDTs dominated the total OHC load for both killer whale dietary clusters, together contributing 87% and 86% of the total OHC burden for seal-eaters and fish-eaters, respectively (Table 1). PCB congeners − 99, − 138, − 153 and − 180 accounted for 67% (for seal-eaters) and 58% (for fish-eaters) of the total ΣPCB load, with PCB-153 contributing the most. The ΣDDTs were dominated by p,p′-DDE, contributing 90% of the total ΣDDTs for the seal-eaters and 92% for fish-eaters. The least contributing were the BFRs (1.5% for seal-eaters and 1.3% for fish-eaters of total OHC load) and OH-metabolites (0.01% for seal-eaters and 0.007% for fish-eaters of total OHC load).

### Influence of sex/age class on pollutant levels

Previous studies have shown age-dependent increases for both OHC and total Hg in killer whales due to age accumulation^34,35^, and lower OHC levels in reproductive females due to maternal offload^17,36–38^. These factors were not possible to be tested in the present study due to a small sample size for adult female individuals in each dietary group (Tables 1, 2), the undetermined sex of five whales, and no possibility to obtain the precise age of the whales. The influence of these factors on the pollutant levels will be a focus for further research as our knowledge of the sampled whales, and their population, increases.

### Implications for dietary ecology

The higher OHC and total Hg levels in seal-eaters indicate a long-term preference for higher trophic prey, in accordance with field observations and stable isotope analysis^14, 15^. The PCB patterns further support long-term preferences for marine mammals, with the seal-eating killer whales having a higher proportion of hexaCBs, heptaCBs, and higher CBs than the fish-eating killer whales (Welch’s t-test, *t*_28_ = 5.38, *p* = 0.001; Supplementary Fig. S1). Higher-chlorinated PCBs are often found in marine mammals relative to fish^39,40^, and higher proportions of hexaCBs and heptaCBs have been found in marine mammal-eating killer whales compared to fish-eating whales in the Northeast Pacific^8,9^, and in seal-eating whales in Greenland compared to fish-eating whales in the Faroe Islands^17^.

There is a 20-fold difference in median ΣPCB levels between two killer whale populations inhabiting the same area of the northeast Pacific, one of which solely eats marine mammals and the other fish^9,41^. This is a larger difference than the five-fold difference between median ΣPCB levels in fish and seal-eating killer whales in our study, and thus may indicate support for previous studies proposing a mixed diet for the seal-eating whales, in which fish prey is an equal or secondary food source to seal prey^14^. It is theorised that some killer whales in Norway diversified to eating seals as a response to the collapse of the herring fisheries in the 1970s^15^. Future changes in prey availability could mean that more killer whales switch to eating seal and/or incorporate a higher proportion of seals in the diet, with subsequent health and population effects. Our results illustrate that differentiations in dietary preferences need to be accounted for in effective risk assessment, management and conservation of the environment.

## Methods

### Sampling

Killer whale biopsy samples of skin and blubber from 38 individuals were collected year-round from August 2017 to July 2018 in northern Norway. All whales were sampled according to relevant guidelines and regulations, and conducted under the permit FOTS-ID 10176 issued by Mattilsynet (the Norwegian Food Safety Authority, report nr. 2016/179856). Details of seasonal sampling locations, stable isotope dietary descriptors and classification of sampled individuals are described in a previous study^14^. In the current study, total Hg was analysed in skin from all individuals (*n* = 38), whereas organohalogen contaminants (OHC) was analysed in blubber of 31 individuals due to insufficient blubber for the remaining 7 individuals.

### OHC analysis

OHC analysis was conducted at the Laboratory of Environmental Toxicology at the Norwegian University of Life Sciences, Oslo, Norway. We analysed a total of 83 OHCs: 49 organochlorines (OCs), including 34 PCBs and 15 organochlorine pesticides (OCPs), 18 brominated flame retardants (BFRs), including newer and unregulated compounds, and 16 hydroxylated metabolites (OH-metabolites) of PCBs and polybrominated diphenylethers (PBDEs). A full list of analysed compounds can be found in Supplementary Table S1.

We analysed OCs and BFRs using a multicomponent method, first described in 1978^42^, and since modified for a range of compounds and biological matrices^43–46^. The analysis of the OH-metabolites was conducted according to previously published methods^47,48^. An outline of the method is described in the Supplementary Information. Reported concentrations were blank corrected based on the average concentration detected within blank samples. The limit of detection (LOD) was defined as three times the average noise in chromatograms, and ranged from 0.40 to 11.10 ng/g w.w. for OCs, 0.012 to 0.362 ng/g w.w. for BFRs and 0.013 to 0.040 ng/g w.w. for OH-metabolites (see Supplementary Table S2). Internal reference materials for OCs and BFRs (contaminated seal blubber, MTref01) and OH-metabolites (contaminated seal blood, MTref03) were also extracted in conjunction with sample material to assess method performance. Internal standard recoveries are listed in Supplementary Table S2.

### Hg analysis

We analysed total Hg by atomic absorption spectrometry at the University of Oslo, using a Direct Mercury Analyser (DMA-80, Milestone Srl, Soirsole, Italy). Killer whale skin samples were freeze dried in a Leybold-Heraeus GT2 freeze dryer with a Leybold Vakuum GmbH vacuum pump (Leybold, Cologne, Germany) and then homogenised to a fine powder using an agate pestle and mortar. Approximately 0.002 g of killer whale skin were analysed in parallel with sample blanks and certified reference material (DORM-4, fish protein; DOLT-5, dogfish liver, National Research Council, Ottawa, Canada). If enough material, samples were analysed in duplicates to ensure precision of measurements and the arithmetic mean value used. Average recoveries of the certified reference materials were within 10% of the reported values. The detection limit of the instrument was 0.05 ng mercury.

### Data treatment

We included OHC compounds found in levels above the instrument’s LOD in a minimum of 65% of the individual whale samples for statistical analysis (see Supplementary Table S1, Supporting Information for pollutants excluded). For individual concentrations below the LOD, we imputed left-censored data by replacing missing values with a random number between 0 and the LOD assuming a beta distribution (α = 5, β = 1) to retain the pattern of the dataset. In total, 95 values below the LOD were replaced, representing 6.52% of the OHC dataset. All total Hg samples were above the LOD.

We defined the ΣPCBs as the sum of all 28 PCB congeners detected in more than 65% of the whale samples (PCB-28, -66, -74, -87, -99, -101, 105, -110, -114, -118, -128, -137, -138, -141, -149, -151, -153, -156-, 157, 170, -180, -183, -187, -189, 194, -196, -206, -209). The definition for ΣPCBs varies within killer whale literature, with some studies analysing only a few core PCB congeners^35^, some all 209 of the possible congeners^36^, and others not providing a definition (e.g. for thresholds for possible health effects^7^). There will therefore inevitably be some errors in comparisons. However, since the ΣPCBs in killer whales is dominated by a few commonly reported congeners, typically PCB-153 and -138^16,37^, it is unlikely that the inclusion of other minor constituents will have a major influence on the total load. PCBs were further grouped according to the number of chlorine substitutions per molecule, i.e. homologue group to compare the pattern of PCBs. ΣDDTs was defined as the sum of p,p′-DDT, p,p′-DDD and p,p′-DDE, the ΣPBDEs as the sum of BDE-28, -47, -99, -100, -153 and -154 and the sum of chlordanes (ΣCHLs) as the sum of oxychlordane, *trans*-chlordane, *cis*-chlordane, *trans*-nonachlor and *cis*-nonachlor.

### Statistical analyses

Statistical analyses were performed using R v. 3.4.1^49^. The significance level was to set to α = 0.05, except in cases where the value was adjusted due to multiple testing, and was two-tailed. In addition to visual inspection, normality was tested using the Shapiro–Wilk’s test^50^ and homogeneity of variance by Levene’s test^51^ using the R package *car*^52^.

### Whale dietary groups

The dietary groups used in this study are based on a previous study, which used stable isotope values inputted into a Gaussian mixture model to assign sampled individuals to two fish-eating groups: *Herring-eaters* and *Lumpfish-eaters* and one mammal-eating group *Seal-eaters*^14^*.* The three dietary groups were characterised by disparate, non-overlapping isotopic niches that were consistent with predatory field observations. The seal-eating group was defined by higher δ^15^N values than the two fish-eating groups.

We found that the herring and lumpfish-eating killer whales did not differ in either their OHC levels (Tukey’s HSD: *p* = 0.49) or total Hg levels (pairwise Welch’s t-test: *p* = 0.67). In this study, we thus combined the dietary groups *Herring-eaters* and *Lumpfish-eaters* into the group *Fish-eaters*, to enable easier comparison to the seal-eating killer whales.

We then used Welch’s t-test to compare the ΣPCB levels in the seal-eating and fish-eating dietary groups (using a log10 transformation), and to compare the total Hg levels in the skin between the two dietary groups.

#### OHC dataset

We used multivariate analysis to compare and visualise the differences in all the OHCs between the dietary groups, age and sex classes using the *vegan* package in R^53^. Principle Component Analysis (PCA) was used to visualise the main structure of the data: reducing the dimensions to two new, uncorrelated, latent variables termed principle components 1 and 2 (PC1 and PC2). We log-10 transformed contaminant levels to ensure normality and homogeneity of variance, and the presence of any influential outliers were checked by the Cook’s distance test. Redundancy Analysis (RDA) was used to extract and summarise the variation in the OHC levels constrained, and thereby explained, by a set of explanatory variables^54^. Significant associations between response variables and the explanatory variables were identified by an RDA based forward model selection, followed by a Monte Carlo forward permutation test (1,000 unrestricted permutations). The samples’ scores along PC1 were subject to one-way Analysis of Variance (ANOVA) followed by Tukey’s honestly significant difference post hoc test (Tukey’s HSD) to analyse differences between the three dietary groups. PC1 scores were also used to evaluate correlation to total Hg levels in the skin using a Spearman’s rank correlation test. Absolute concentrations were subject to PCA with lipid % as a covariate, after checking its significance using RDA, as lipid normalising data in inferential statistics can often lead to misleading conclusions^55^.

We lipid-normalised OHC values when comparing levels to threshold values for toxicity or other killer whale populations, and used the geometric mean as the average for each dietary group to reflect the log normal distribution of the data. In accordance with convention, efforts were made to only compare adult males with other worldwide populations, as reproductive female whales are known to transfer a substantial portion of their OHC burden to their calves^35,36,38^. In any case of comparison, similar metrics were compared (i.e. arithmetic mean, geometric mean, median) and all variables kept similar (i.e. sex, age, biopsy/stranded animals). We make the assumption in this study that the killer whales sampled in 2002 in Norway were fish-eaters for the following reasons: firstly, the whales were sampled on herring overwintering grounds, feeding on herring, and photographs were taken of five of the eight adults sampled and were identified as herring-eaters from previous field observations^16^. Secondly, the PCB pattern in the sampled whales showed 76% of ΣPCBs higher chlorinated congeners (hexaCBs or higher), which is more similar to the fish-eaters from our study (80% higher chlorinated congeners) than the seal-eaters (87% higher chlorinated congeners). Thirdly, the upper 95% confidence range of all pollutants reported in the 2002 killer whales falls below both the geometric and arithmetic mean values for seal-eaters from this study.

#### Total Hg dataset

The normal distribution of the data within each dietary group meant we used the arithmetic mean as an average. The three dietary groups (*Herring-eaters, Lumpfish-eaters and Seal-eaters)* were compared using a pairwise Welch’s t-test with a Benjamini–Hochberg False Discovery Rate correction to adjust for multiple testing. Because we found no difference between the *Herring-eaters* and *Lumpfish-eaters* (*p* = 0.67), we combined these two groups to a group called “*Fish-eaters*” for easier comparison with the seal-eaters. The total Hg levels in the skin of the two groups, *Fish-eaters* and *Seal-eaters* were compared using Welch’s t-test.

There is a strong positive correlation between Hg levels in the skin and liver in toothed whales, and this can be used to compare Hg levels measured in skin with hepatic toxicity threshold values^56–58^. To extrapolate to liver from skin in our samples, we chose an equation based on a model using concentrations in the liver (Hg_liver_ μg/g w.w) and skin (Hg_skin_ μg/g w.w) of bottlenose dolphins (*Tursiops truncatus*) (Eq. 1)^58^. We converted dry weight to wet weight using the water content for each individual whale measured during freeze drying.

1 $$\ln \left( {Hg_{liver} } \right) = 1.6124 \times \ln \left( {Hg_{skin} } \right) + 2.0346$$

When comparing Hg concentrations to other worldwide populations, both male and female whales were included. This was due to a lack of information of sex in one of the populations for comparisons and because killer whales are unlikely to pass on Hg burdens to calves^5,59^.

## Supplementary information

Supplementary file1 (PDF 699 kb)

Supplementary file2 (XLSX 31 kb)

## Data Availability

All data is available in the main text or the Supplementary information.

## References

[1] Letcher RJ (2010). Exposure and effects assessment of persistent organohalogen contaminants in Arctic wildlife and fish. Sci. Total Environ..

[2] Dietz R (2013). What are the toxicological effects of mercury in Arctic biota?. Sci. Total Environ..

[3] Dietz R (2013). Three decades (1983–2010) of contaminant trends in East Greenland polar bears (*Ursus maritimus*). Part 1: Legacy organochlorine contaminants. Environ. Int..

[4] Tanabe S (1988). PCB problems in the future: Foresight from current knowledge. Environ. Pollut..

[5] AMAP. Arctic Monitoring and Assessment Programme: AMAP Assessment Report-Biological effects of contaminants on Arctic wildlife and fish, Tromsø, Norway (2018).

[6] Desforges J-P (2018). Predicting global killer whale population collapse from PCB pollution. Science.

[7] Dietz R (2019). Current state of knowledge on biological effects from contaminants on arctic wildlife and fish. Sci. Total Environ..

[8] Herman DP (2005). Feeding ecology of Eastern North Pacific killer whales *Orcinus orca* from fatty acid, stable isotope, and organochlorine analyses of blubber biopsies. Mar. Ecol. Prog. Ser..

[9] Buckman AH (2011). PCB-associated changes in mRNA expression in killer whales (*Orcinus orca*) from the NE Pacific ocean. Environ. Sci. Technol..

[10] Ruus A (2015). Methylmercury biomagnification in an Arctic pelagic food web. Environ. Toxicol. Chem..

[11] Tokar EJ, Boyd WA, Freedman JH, Waalks MP, Klaasen CD, Watkins JB (2015). Toxic effects of metals. Casarett & Doull’s Essentials of Toxicology.

[12] Bisther A, Vongraven D, Blix AS, Walløe L, Ulltang Ø (1995). Studies of the social ecology of Norwegian killer whales (*Orcinus orca*). Whales, Seals, Fish and Man.

[13] Similä T, Holst JC, Christensen I (1996). Occurrence and diet of killer whales in northern Norway: Seasonal patterns relative to the distribution and abundance of Norwegian spring-spawning herring. Can. J. Fish. Aquat. Sci..

[14] Jourdain E (2020). Isotopic niche differs between seal and fish-eating killer whales (*Orcinus orca*) in northern Norway. Ecol. Evol..

[15] Jourdain E, Vongraven D, Bisther A, Karoliussen R (2017). First longitudinal study of seal-feeding killer whales (*Orcinus orca*) in Norwegian coastal waters. PLoS ONE.

[16] Wolkers H, Corkeron PJ, Van Parijs SM, Simila T, Van Bavel B (2007). Accumulation and transfer of contaminants in killer whales (*Orcinus orca*) from Norway: Indications for contaminant metabolism. Environ. Toxicol. Chem..

[17] Pedro S (2017). Blubber-depth distribution and bioaccumulation of PCBs and organochlorine pesticides in Arctic-invading killer whales. Sci. Total Environ..

[18] Desforges J-P (2016). Immunotoxic effects of environmental pollutants in marine mammals. Environ. Int..

[19] Mori C, Morsey B, Levin M, Nambiar PR, De Guise S (2006). Immunomodulatory effects of in vitro exposure to organochlorines on T-cell proliferation in marine mammals and mice. J. Toxicol. Environ. Health Part A.

[20] Kannan K, Blankenship AL, Jones PD, Giesy JP (2000). Toxicity reference values for the toxic effects of polychlorinated biphenyls to aquatic mammals. Hum. Ecol. Risk Assess. Int. J..

[21] Jepson PD (2016). PCB pollution continues to impact populations of orcas and other dolphins in European waters. Sci. Rep..

[22] Luster M (1993). Risk assessment in immunotoxicology. II. Relationships between immune and host resistance tests. Fundam. Appl. Toxicol..

[23] Hall AJ (2018). Predicting the effects of polychlorinated biphenyls on cetacean populations through impacts on immunity and calf survival. Environ. Pollut..

[24] Helle E, Olsson M, Jensen S (1976). DDT and PCB levels and reproduction in ringed seal from the Bothnian Bay. AMBIO A J. Hum. Environ..

[25] Kuningas S, Similä T, Hammond PS (2014). Population size, survival and reproductive rates of northern Norwegian killer whales (*Orcinus orca*) in 1986–2003. J. Mar. Biol. Assoc. U.K..

[26] Rawson AJ, Patton GW, Hofmann S, Pietra GG, Johns L (1993). Liver abnormalities associated with chronic mercury accumulation in stranded Atlantic bottlenose dolphins. Ecotoxicol. Environ. Saf..

[27] Ronald K, Tessaro SV, Uthe JF, Freeman HC, Frank R (1977). Methylmercury poisoning in the harp seal (*Pagophilus groenlandicus*). Sci. Total Environ..

[28] Zanuttini C (2019). High pollutant exposure of bottlenose dolphins from the largest European community in the English Channel. Sci. Rep..

[29] Frodello JP, Romeo M, Viale D (2000). Distribution of mercury in the organs and tissues of five toothed-whale species of the Mediterranean. Environ. Pollut..

[30] Wójtowicz AK, Kajta M, Gregoraszczuk EŁ (2007). DDT- and DDE-induced disruption of ovarian steroidogenesis in prepubertal porcine ovarian follicles: A possible interaction with the main steroidogenic enzymes and estrogen receptor beta. J. Physiol. Pharmacol..

[31] Hamers T (2006). In vitro profiling of the endocrine-disrupting potency of brominated flame retardants. Toxicol. Sci..

[32] United Nations Environment Programme. *Stockholm convention on persistent organic pollutants (POPs). Geneva, Switzerland: United Nations Environment Programme*. (2001). https://www.wipo.int/edocs/lexdocs/treaties/en/unep-pop/trt_unep_pop_2.pdf.

[33] Rigét F (2019). Temporal trends of persistent organic pollutants in Arctic marine and freshwater biota. Sci. Total Environ..

[34] Endo T, Kimura O, Hisamichi Y, Minoshima Y, Haraguchi K (2006). Age-dependent accumulation of heavy metals in a pod of killer whales (*Orcinus orca*) stranded in the northern area of Japan. Chemosphere.

[35] Krahn MM (2009). Effects of age, sex and reproductive status on persistent organic pollutant concentrations in ‘Southern Resident’ killer whales. Mar. Pollut. Bull..

[36] Ross PS, Ellis GM, Ikonomou MG, Barrett-Lennard LG, Addison RF (2000). High PCB concentrations in free-ranging Pacific killer whales, *Orcinus orca*: Effects of age, sex and dietary preference. Mar. Pollut. Bull..

[37] Ylitalo GM (2001). Influence of life-history parameters on organochlorine concentrations in free-ranging killer whales (*Orcinus orca*) from Prince William Sound, AK. Sci. Total Environ..

[38] Haraguchi K, Hisamichi Y, Endo T (2009). Accumulation and mother-to-calf transfer of anthropogenic and natural organohalogens in killer whales (*Orcinus orca*) stranded on the Pacific coast of Japan. Sci. Total Environ..

[39] Hoekstra PF (2003). Trophic transfer of persistent organochlorine contaminants (OCs) within an Arctic marine food web from the southern Beaufort-Chukchi Seas. Environ. Pollut..

[40] Sobek A (2010). A comparison of PCB bioaccumulation factors between an Arctic and a temperate marine food web. Sci. Total Environ..

[41] Ford JKB (1998). Dietary specialization in two sympatric populations of killer whales (*Orcinus orca*) in coastal British Columbia and adjacent waters. Can. J. Zool..

[42] Brevik EM (1978). Gas chromatographic method for the determination of organochlorine pesticides in human milk. Bull. Environ. Contam. Toxicol..

[43] Bernhoft A, Skaare JU (1994). Levels of selected individual polychlorinated biphenyls in different tissues of harbour seals (*Phoca vitulina*) from the southern coast of Norway. Environ. Pollut..

[44] Andersen M (2001). Geographic variation of PCB congeners in polar bears (*Ursus maritimus*) from Svalbard east to the Chukchi Sea. Polar Biol..

[45] Polder A (2014). Levels and patterns of persistent organic pollutants (POPs) in tilapia (*Oreochromis* sp.) from four different lakes in Tanzania: Geographical differences and implications for human health. Sci. Total Environ..

[46] Polder A (2008). Spatial and temporal changes of chlorinated pesticides, PCBs, dioxins (PCDDs/PCDFs) and brominated flame retardants in human breast milk from Northern Russia. Sci. Total Environ..

[47] Løken KB, Lie E, Lundanes E, Skåre JU (2006). Extension of a multicomponent method to include the determination of OH-PCBs and OH-PBDEs in biological matrices. Organohalogen Compd..

[48] Gabrielsen KM (2011). Levels and patterns of hydroxylated polychlorinated biphenyls (OH-PCBs) and their associations with thyroid hormones in hooded seal (*Cystophora cristata*) mother–pup pairs. Aquat. Toxicol..

[49] R Development Core Team. *R: A Language and Environment for Statistical Computing*. R Foundation for Statistical Computing, Vienna. https://www.R-project.org/. (2019).

[50] Shapiro SS, Wilk MB (1965). An analysis of variance test for normality (complete samples). Biometrika.

[51] Levene H (1960). A robust approximate confidence-interval for components of variance. Ann. Math. Stat..

[52] Fox J, Weisberg S (2011). An R Companion to Applied Regression.

[53] Oksanen, J. *et al.* vegan: Community Ecology Package. R package version 2.5-4. (2019).

[54] Palmer MW, McGlinn DJ, Westerberg L, Milberg P (2008). Indices for detecting differences in species composition: Some simplifications of RDA and CCA. Ecology.

[55] Hebert CE, Keenleyside KA (1995). To normalize or not to normalize? Fat is the question. Environ. Toxicol. Chem..

[56] Aubail A (2013). Use of skin and blubber tissues of small cetaceans to assess the trace element content of internal organs. Mar. Pollut. Bull..

[57] Cáceres-Saez I, Goodall RNP, Dellabianca NA, Cappozzo HL, Guevara SR (2015). The skin of Commerson’s dolphins (*Cephalorhynchus commersonii*) as a biomonitor of mercury and selenium in Subantarctic waters. Chemosphere.

[58] Stavros H-CW (2011). Correlation and toxicological inference of trace elements in tissues from stranded and free-ranging bottlenose dolphins (*Tursiops truncatus*). Chemosphere.

[59] Endo T (2006). Distribution of total mercury, methyl mercury and selenium in pod of killer whales (*Orcinus orca*) stranded in the northern area of Japan: Comparison of mature females with calves. Environ. Pollut..

