# Supplementary material for: Preying on seals pushes killer whales from Norway above pollution effects thresholds
Source: Sci Rep. 2020 Jul 17;10:11888. doi: 10.1038/s41598-020-68659-y (PMC7368030; doi:10.1038/s41598-020-68659-y)
Supplement: Supplementary file 1 — Supplementary file1 (PDF 699 kb) [file 41598_2020_68659_MOESM1_ESM.pdf]

Supplementary Information for:

Preying on seals pushes killer whales from Norway above pollution effects thresholds

Clare Andvik[1], Eve Jourdain[2], Anders Ruus[1,3], Jan L. Lyche[4], Richard Karoliussen[2], Katrine Borgå[1*]

[1] Department of Biosciences, University of Oslo, NO-0316 Oslo, Norway.

[2] Norwegian Orca Survey, Daniel Olaisensvei 6, NO-8480 Andenes, Norway.

[3] Norwegian Institute of Water Research, Gaustadalléen 21, NO-0349 Oslo, Norway

[4] Department of Food Safety and Infection Biology, Norwegian University of Life Sciences, P.O. Box 5003, Campus Adamstuen, NO-1432 Ås, Norway

*Correspondence to: katrine.borga@ibv.uio.no

Supplementary Figure S1:

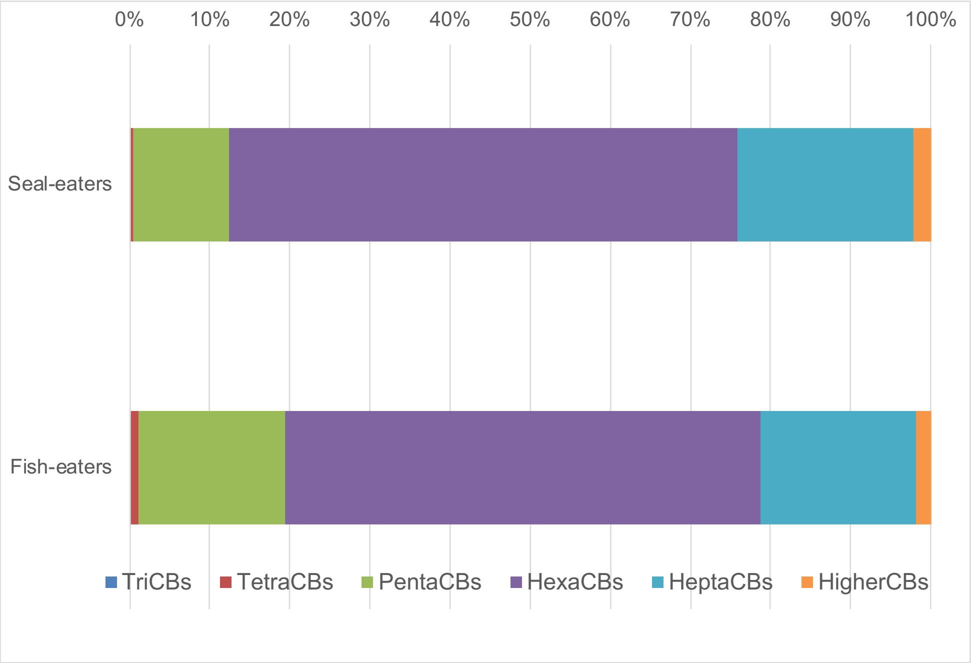

Relative contribution (%) of each homologue group of polychlorinated biphenyls (PCBs), defined according to number of chlorines, to the total PCB load (ΣPCBs) in the blubber of two dietary groups of killer whale (*Orcinus orca*) collected July 2017–July 2018 in northern Norway. Seal-eaters: n = 7; fish-eaters: n = 24). ΣPCBs is defined as the sum of PCB congeners detected in over 65% of whale samples: PCB-28, -66, -74, -87, -99, -101, -105, -110, -114, -118, -128, -137, -138, -141, -149, -151, -153, -156-, 157, 170, -180, -183, -187, -189, 194, -196, -206, -209

Supplementary Methods: Organohalogen contaminant method

Approximately 0.5 g of killer whale blubber was spiked with internal standards: PCB-29, -112 and -207 (Ultra Scientific, North Kingstown, RI), BDE-77, -119, -181 and -[$^{13}C_{12}$]-209 (Cambridge Isotope Laboratories, Tewksbury, MA) and 4'-OH-[$^{13}C_{12}$]-CB-159 and 4-OH-[$^{13}C_{12}$]-CB-187 (Wellington Laboratories Inc., Guelph, Canada), homogenised using an Ultra Turrax® (IKA T25, Labortechnik, Wasserburg, Germany), and the sample homogenate extracted twice using cyclohexane and acetone. The supernatants from each step were combined, and 20% of the combined supernatant aliquoted into a vial for gravimetric lipid determination to calculate lipid %. Sample extracts were cleaned with concentrated sulphuric acid to remove fat and lipid residues. OH-metabolites were extracted using potassium hydroxide and sulphuric acid, and peak-tailing during gas chromatography (GC) prevented by replacing hydroxyl groups with acetyl groups using 1:1 pyridine: acetic anhydride. Sample extracts were evaporated and quantitively transferred to GC glasses, and OCs, BFRs and OH-metabolites analysed separately. Instrumental methods and equipment set-up has been described previously,[46,48] with the following modifications: in quantifying OCs the constant flow of the hydrogen carrier gas was increased to 1.3 mL/min. and the final holding time at 275 °C was increased to 12 min, making the total run time 71.6 min.

The laboratory is accredited by the Norwegian Accreditation for the determination of OCs and BFRs in biological matrices of animal origin according to the requirements of NS-EN ISO/IEC 17025:2005 (Test 137). The laboratory is accredited for all analytes in the present study with the following exceptions: BDE-196, -202, -206, -207, -208, -209, PBT, PBEB, DPTE, HBB and OH-metabolites of PCBs and PBDEs. The method for determining non-accredited analytes was performed and validated following the same principles as the accredited standard (NS- EN ISO/IEC 17025).

Supplementary Data is raw data provided in a separate excel file

Supplementary Table S1. Summary of pollutant datasets used in statistical analyses. Pollutants were considered if they were detected in 65% or more of the whale blubber samples analysed.

	Pollutant group	Included	Excluded
**OHCs (Lipophilic)** ***n* = 31**	PCBs (N = 34)	PCB-28, -66, -74, -87, -99, -101, -105, -110, -114, -118, -128, -137, -138, -141, -149, -151, -153, -156, -157, -170, -180, -183, -187, -189, -194, -196, -206, -209	PCB-31, -47, -52, -56, -136, -199
	OCPs (N = 15)	p,p'-DDT, p,p'-DDD, p,p'-DDE, HCB, β-HCH, oxychlordane, *trans*-chlordane, *cis*-chlordane, *trans*-nonachlor, *cis*-nonachlor, Mirex	o,p'-DDT, o,p'-DDD, α-HCH, γ-HCH
	BFRs (N = 18)	BDE-28, -47, -99, -100, -153, -154, HBB	BDE-183, -196, -202, -206, -207, -208, -209, HBCDD, PBT, PBEB, DPTE
	OH-metabolites (N = 16)	4'-OH-BDE49	4'-OH-PCB106, 4-OH-PCB107, 4'-OH-PCB108, 3-OH-PCB118, 4'-OH-PCB130, 3'-OH-PCB138, 4-OH-PCB146, 4'-OH-PCB159, 4'-OH-PCB172, 3'-OH-PCB180, 4-OH-PCB187, 4-OH-BDE42, 3-OH-BDE47, 6-OH-BDE47
**Mercury (Protein-associated)** ***n* = 38**	Total mercury (N = 1)	Total mercury	

Supplementary Table S2. Summary abbreviations, IUPAC name, limit of detection, recovery, and % found in all whale samples for all organohalogen contaminants analysed

Pollutant abbreviation	IUPAC name	Limit of detection	Recovery	% found in whales
PCB-28	2,4-dichloro-1-(4-chlorophenyl)benzene	0.44	102	67.7
PCB-31	1,4-dichloro-2-(4-chlorophenyl)benzene	0.44	97	0
PCB-47	2,4-dichloro-1-(2,4-dichlorophenyl)benzene	11.10	106	45.1
PCB-52	1,4-dichloro-2-(2,5-dichlorophenyl)benzene	8.83	107	61.2
PCB-56	1,2-dichloro-3-(3,4-dichlorophenyl)benzene	1.33	101	0
PCB-66	1,2-dichloro-4-(2,4-dichlorophenyl)benzene	0.80	99	83.8
PCB-74	1,2,4-trichloro-5-(4-chlorophenyl)benzene	1.68	98	87
PCB-87	1,2,3-trichloro-4-(2,5-dichlorophenyl)benzene	1.42	102	80.6
PCB-99	1,2,4-trichloro-5-(2,4-dichlorophenyl)benzene	2.83	102	100
PCB-101	1,2,4-trichloro-5-(2,5-dichlorophenyl)benzene	2.30	100	96.8
PCB-105	1,2,3-trichloro-4-(3,4-dichlorophenyl)benzene	0.45	102	100
PCB-110	1,2,4-trichloro-3-(3,4-dichlorophenyl)benzene	1.60	102	77.4
PCB-114	1,2,3,4-tetrachloro-5-(4-chlorophenyl)benzene	0.45	103	77.4
PCB-118	1,2,4-trichloro-5-(3,4-dichlorophenyl)benzene	0.45	102	96.8
PCB-128	1,2,3-trichloro-4-(2,3,4-trichlorophenyl)benzene	0.45	102	100
PCB-136	1,2,4-trichloro-3-(2,3,6-trichlorophenyl)benzene	6.90	102	54.8
PCB-137	1,2,3,4-tetrachloro-5-(2,4-dichlorophenyl)benzene	0.45	104	100
PCB-138	1,2,3-trichloro-4-(2,4,5-trichlorophenyl)benzene	0.71	101	100
PCB-141	1,2,3,4-tetrachloro-5-(2,5-dichlorophenyl)benzene	0.45	104	87
PCB-149	1,2,4-trichloro-3-(2,4,5-trichlorophenyl)benzene	0.89	100	96.8
PCB-151	1,2,4,5-tetrachloro-3-(2,5-dichlorophenyl)benzene	0.45	102	96.8
PCB-153	1,2,4-trichloro-5-(2,4,5-trichlorophenyl)benzene	0.71	101	100
PCB-156	1,2,3,4-tetrachloro-5-(3,4-dichlorophenyl)benzene	0.53	103	96.8
PCB-157	1,2,3-trichloro-4-(3,4,5-trichlorophenyl)benzene	0.45	103	96.8
PCB-170	1,2,3,4-tetrachloro-5-(2,3,4-trichlorophenyl)benzene	0.45	101	100
PCB-180	1,2,3,4-tetrachloro-5-(2,4,5-trichlorophenyl)benzene	0.45	101	100
PCB-183	1,2,3,5-tetrachloro-4-(2,4,5-trichlorophenyl)benzene	0.45	104	100
PCB-187	1,2,4,5-tetrachloro-3-(2,4,5-trichlorophenyl)benzene	0.45	103	100
PCB-189	1,2,3,4-tetrachloro-5-(3,4,5-trichlorophenyl)benzene	0.45	101	83.8
PCB-194	1,2,3,4-tetrachloro-5-(2,3,4,5-tetrachlorophenyl)benzene	0.45	103	100
PCB-196	1,2,3,4-tetrachloro-5-(2,3,4,6-tetrachlorophenyl)benzene	0.45	100	100
PCB-199	1,2,3,4-tetrachloro-5-(2,3,5,6-tetrachlorophenyl)benzene	0.45	101	51.6
PCB-206	1,2,3,4,5-pentachloro-6-(2,3,4,5-tetrachlorophenyl)benzene	0.45	101	83.8
PCB-209	1,2,3,4,5-pentachloro-6-(2,3,4,5,6-pentachlorophenyl)benzene	0.40	97	93.5
HCB	1,2,3,4,5,6-hexachlorobenzene	0.45	106	100

α-HCH	α-1,2,3,4,5,6-hexachlorocyclohexane	0.90	103	9.6
β-HCH	β-1,2,3,4,5,6-hexachlorocyclohexane	1.24	100	74.1
γ-HCH	γ-1,2,3,4,5,6-hexachlorocyclohexane	1.15	103	3.3
Oksyklordan	1,5,6,8,9,10,11,11-octachloro-4-oxatetracyclo[6.2.1.0^{2,7}.0^{3,5}]undec-9-ene	0.45	65	100
trans-Klordan	(1S,2*S*,3*R*,4*R*,6*S*,7R)-1,3,4,7,8,9,10,10-octachlorotricyclo[5.2.1.0^{2,6}]dec-8-ene	0.45	109	83.8
cis-Klordan	(1*R*,2*R*,3*R*,4*S*,6*S*,7*S*)-1,3,4,7,8,9,10,10-octachlortricyclo[5.2.1.0^{2,6}]dec-8-ene	0.45	108	96.7
trans-Nonaklor	(1*S*,2*R*,3*S*,5*R*,6*S*,7*R*)-1,3,4,5,7,8,9,10,10-nonachlortricyclo[5.2.1.0^{2,6}]dec-8-ene	0.45	108	100
cis-Nonaklor	(1*S*,2*R*,3*R*,5*S*,6*R,*7*R*)-1,3,4,5,7,8,9,10,10-nonachlortricyclo[5.2.1.0^{2,6}]dec-8-ene	0.45	104	93.5
p,p'-DDE	1-chloro-4-[2,2,2-trichloro-1-(4- [illegible]	4.24	103	100
o,p'-DDD	1-chloro-2-[2,2,2-trichloro-1-(4- [illegible]	2.30	109	51.6
p,p'-DDD	1-chloro-2-[2,2-dichloro-1-(4-chlorophenyl)ethyl]benzene	8.22	104	87
o,p'-DDT	1-chloro-4-[2,2-dichloro-1-(4-chlorophenyl)ethyl]benzene	6.00	108	61.2
p,p'-DDT	1-chloro-4-[2,2-dichloro-1-(4- [illegible]	1.33	108	80.6
Mirex	1,2,3,4,5,5,6,7,8,9,10,10-dodecachloropentacyclo[5.3.0.0^{2,6}.0^{3,9}.0^{4,8}]decane	0.62	98	90.3
BDE-28	2,4-dibromo-1-(4-bromophenoxy)benzene	0.017	92	100
BDE-47	2,4-dibromo-1-(2,4-dibromophenoxy)benzene	0.030	90	100
BDE-99	1,2,4-tribromo-5-(2,4-dibromophenoxy)benzene	0.034	97	100
BDE-100	1,3,5-tribromo-2-(2,4-dibromophenoxy)benzene	0.040	87	100
BDE-153	1,2,4-tribromo-5-(2,4,5-tribromophenoxy)benzene	0.030	96	100
BDE-154	1,2,4-tribromo-5-(2,4,6-tribromophenoxy)benzene	0.030	92	100
BDE-183	1,2,3,5-tetrabromo-4-(2,4,5-tribromophenoxy)benzene	0.085	84	22.58
BDE-196	1,2,3,4-tetrabromo-5-(2,3,4,6-tetrabromophenoxy)benzene	0.362	180	3.3
BDE-202	1,2,4,5-tetrabromo-3-(2,3,5,6-tetrabromophenoxy)benzene	0.030	216	45.1
BDE-206	1,2,3,4,5-pentabromo-6-(2,3,4,5- [illegible]	0.110	117	16.12
BDE-207	1,2,3,4,5-pentabromo-6-(2,3,4,6- [illegible]	0.055	126	19.3
BDE-208	1,2,3,4,5-pentabromo-6-(2,3,5,6- [illegible]	0.063	115	3.3
BDE-209	1,2,3,4,5-pentabromo-6-(2,3,4,5,6- [illegible]	0.030	100	25.8
HBCDD	1,2,5,6,9,10-hexabromocyclododecane	0.122	54	58
PBT	1,2,3,4,5-pentabromo-6-methylbenzene	0.012	94	58
PBEB	1,2,3,4,5-pentabromo-6-ethylbenzene	0.014	92	3.2
DPTE	(1,3,5-tribrom-2-(2,3 dibrompropoxy)benzene	0.027	95	0
HBB	1,2,3,4,5,6-hexabromobenzene	0.017	94	100
4'-OH-CB106	4'-OH-2',3,3',4',5'-pentachlorobiphenyl	0.018	63	3.2
4-OH-CB107	4-OH-2,3,3',4',5- pentachlorobiphenyl	0.022	103	22.6
4'-OH-CB108	4'-OH-2',3,3',4',5- pentachlorobiphenyl	0.022	100	6.45
3-OH-CB118	3-OH-2,3',4,4',5- pentachlorobiphenyl	0.027	115	3.2
4'-OH-CB130	4'-OH-2,2',3,3',4',5-hexachlorobiphenyl	0.027	104	0
3'-OH-CB138	3'-OH-2,2',3',4,4',5- hexachlorobiphenyl	0.036	105	0

4-OH-CB146	4-OH-2,2',3,4',5,5'- hexachlorobiphenyl	0.022	102	0
4'-OH-CB159	4'-OH-2',3,3',4',5,5'- hexachlorobiphenyl	0.013	93	9.6
4'-OH-CB172	4'-OH-2,2',3,3',4',5,5'- heptachlorobiphenyl	0.013	86	0
3'-OH-CB180	3'-OH-2,2',3',4,4',5,5'- heptachlorobiphenyl	0.013	84	0
4-OH-CB187	4-OH-2,2',3,4',5,5',6- heptachlorobiphenyl	0.027	77	6.45
4-OH-BDE42	4-OH-2,2',3,4'-tetrabromodiphenyl ether	0.040	47	3.2
3-OH-BDE47	3-OH-2,2',4,4'-tetrabromodiphenyl ether	0.027	61	3.2
6-OH-BDE47	6-OH-2,2',4,4'-tetrabromodiphenyl ether	0.027	107	45.1
4'-OH-BDE49	4'-OH-2,2',4,5'-tetrabromodiphenyl ether	0.040	82	83.8
2'-OH-BDE68	2'-OH-2,3',4,5'-tetrabromodiphenyl ether	0.027	91	6.45

Supplementary Table S3: Summary of lipid-normalised concentrations (µg/g) of organohalogen contaminants expressed in all sampled killer whales (*n* = 31)

OHC group		Seal-eaters All (*n* = 7)	Seal-eaters Adult male (*n* = 4)	Seal-eaters Adult female (*n* = 2)	Seal-eaters UNK[f] (*n* = 1)	Fish-eaters All (*n* = 24)	Fish-eaters Adult male (*n* = 17)	Fish-eaters Adult female (*n* = 2)	Fish-eaters SA[g] male (*n* = 1)	Fish-eaters SA[g] UNK (*n* = 2)	Fish-eaters UNK (*n* = 2)
**ΣPCBs[a]**	µ ± SD	56.0 ± 37.8	58.9 ± 35.9	64.6 ± 60.6	26.9	16.7 ± 18.3	20.7 ± 20.4	4.07 ± 2.52	6.87	6.59 ± 3.53	10.6 ± 9.34
	Geometric mean	45.7	50.7	48.4		10.9	14.2	3.66		6.10	8.30
	Median (range)	48.2 (21.8–108)	53.0 (22.0–108)	64.6 (21.8–107)		9.52 (2.3–77.1)	12.0 (4.03–77.1)	4.07 (2.3–5.85)		6.59 (4.09–9.08)	10.6 (3.96–17.2)
**ΣDDTs[b]**	µ ± SD	28.3 ± 21.1	30.9 ± 16.7	32.9 ± 37.2	8.70	15.3 ± 21.4	19.3 ± 20.4	2.49 ± 1.64	4.60	5.73 ± 3.78	9.39 ± 9.03
	Geometric mean	21.4	27.7	19.9		8.59	11.4	2.20		5.06	6.88
	Median (range)	26.8 (6.67–59.2)	27.5 (14.4–54.2)	32.9 (6.67–59.2)		7.79 (1.33–100)	8.96 (3.04–100)	2.49 (1.33–3.65)		5.73 (3.05–8.40)	9.39 (3.00–15.8)
**ΣCHLs[c]**	µ ± SD	9.78 ± 5.59	10.3 ± 3.75	11.6 ± 10.2	4.06	4.35 ± 5.01	5.31 ± 5.65	1.17 ± 0.964	1.99	1.65 ± 0.874	3.31 ± 2.40
	Geometric mean	8.41	9.69	9.13		2.98	3.80	0.952		1.53	2.84
	Median (range)	9.46 (4.06–18.9)	10.6 (5.54–14.4)	11.7 (4.41–18.9)		2.67 (0.489–23.4)	3.06 (1.52–23.4)	1.17 (0.489–1.85)		1.65 (1.03–2.26)	3.31 (1.61–5.01)
**HCB**	µ ± SD	0.523 ± 0.467	0.389 ± 0.309	0.893 ± 0.824	0.357	0.205 ± 0.0903	0.229 ± 0.0934	0.105 ± 0.0570	0.160	0.260 ± 0.0443	0.186 ± 0.006
	Geometric mean	0.401	0.324	0.650		0.189	0.216	0.0973		0.132	0.186
	Median (range)	0.290 (0.220–0.146)	0.242 (0.220–0.852)	0.873 (0.290–1.45)		0.178 (0.0649–0.519)	0.194 (0.126–0.519)	0.105 (0.0649–0.146)		0.260 (0.105–0.167)	0.186 (0.190–0.182)
**β-HCH**	µ ± SD	0.116 ± 0.090	0.0974 ± 0.0692	0.179 ± 0.149	0.0605	0.0470 ± 0.0369	0.0546 ± 0.0935	0.0186 ± 0.0164	0.0164	0.0260 ± 0.00964	0.0476 ± 0.0167
	Geometric mean	0.0929	0.0827	0.145		0.0378	0.0456	0.0146		0.0251	0.0461
	Median (range)	0.0739 (0.0426–0.284)	0.074 (0.0426–0.199)	0.179 0.0739–0.284		0.0311 (<LOD–0.193)	0.0513 (<LOD–0.193)	0.0186 (<LOD–0.030)		0.0260 (<LOD–0.0328)	0.0476 (<LOD–0.0593)
**Mirex**	µ ± SD	0.295 ± 0.148	0.256 ± 0.179	0.400 ± 0.065	0.237	0.0700 ± 0.0777	0.0842 ± 0.0879	0.0384 ± 0.00479	0.0318	0.0407 ± 0.0158	0.0292 ± 0.0386
	Geometric mean	0.224	0.166	0.398		0.0425	0.0522	0.383		0.0391	0.0104
	Median (range)	0.347 (<LOD–0.446)	0.283 (<LOD–0.437)	0.400 (0.354–0.446)		0.0449 (<LOD–0.350)	0.0478 (<LOD–0.304)	0.0384 (<LOD–0.0417)		0.0407 (<LOD–0.0158)	0.0292 (<LOD–0.0565)
**ΣPBDEs[d]**	µ ± SD	1.57 ± 0.73	1.61 ± 0.39	1.67 ± 1.61	1.21	0.554 ± 0.415	0.491 ± 0.444	0.218 ± 0.172	0.275	0.284 ± 0.182	0.482 ± 0.364
	Geometric mean	1.41	1.58	1.22		0.447	0.553	0.180		0.253	0.407
	Median (range)	1.38 (0.529–2.81)	1.51 (1.27–2.13)	1.67 (0.529–2.81)		0.466 (0.0960–1.79)	0.491 (0.258–1.79)	0.218 (0.0960–0.339)		0.284 (0.155–0.412)	0.482 (0.224–0.739)
**HBB**	µ ± SD	(9.04 ± 2.81)×10[illegible]	(9.15 ± 3.61)$\times 10^{-3}$	(10.1 ± 0.02)$\times 10^{-3}$	6.57 $\times 10^{-3}$	(2.82 ± 3.16)$\times 10^{-3}$	(3.49 ± 3.55)$\times 10^{-3}$	(1.55 ± 0.632)$\times 10^{-3}$	0.729 $\times 10^{-3}$	(0.954 ±0.189) $\times 10^{-3}$	(1.40 ± 1.08)$\times 10^{-3}$
	Geometric mean	8.68 $\times 10^{-3}$	8.64 $\times 10^{-3}$	10.0 $\times 10^{-3}$		1.84 $\times 10^{-3}$	2.27 $\times 10^{-3}$	1.48 $\times 10^{-3}$		0.945 $\times 10^{-3}$	1.17 $\times 10^{-3}$
	Median (range)	9.49 $\times 10^{-3}$ (5.51–14.0)$\times 10^{-3}$	8.55 $\times 10^{-3}$ (5.51–14.0)$\times 10^{-3}$	10.1 $\times 10^{-3}$ (10.1–10.1)$\times 10^{-3}$		1.90 $\times 10^{-3}$ (0.611–14.3)$\times 10^{-3}$	2.18 $\times 10^{-3}$ (0.611–14.3)$\times 10^{-3}$	1.55 $\times 10^{-3}$ (1.10–1.99)$\times 10^{-3}$		0.954 $\times 10^{-3}$ (0.821–1.08)$\times 10^{-3}$	1.40 $\times 10^{-3}$ (0.634–0.217)$\times 10^{-3}$

**4'-OH-BDE49**	μ ± SD	(7.79 ± 5.54)×10[illegible]	(6.96 ± 4.28)$\times10^{-3}$	(10.3 ± 10.6)$\times10^{-3}$	6.06 $\times10^{-3}$	(2.92 ± 3.41)$\times10^{-3}$	(3.81 ± 3.70)$\times10^{-3}$	(0.563 ± 3.62)$\times10^{-3}$	0.729 $\times10^{-3}$	(0.603 ± 0.769)$\times10^{-3}$	(1.08 ± 0.410)$\times10^{-3}$
	Geometric mean	6.20 $\times10^{-3}$	5.83 $\times10^{-3}$	7.07 $\times10^{-3}$		1.45 $\times10^{-3}$	2.20 $\times10^{-3}$	0.464 $\times10^{-3}$		0.261 $\times10^{-3}$	1.05 $\times10^{-3}$
	Median (range)	6.06 $\times10^{-3}$ (2.31–17.8)$\times10^{-3}$	7.27 $\times10^{-3}$ (2.31–11.0)$\times10^{-3}$	10.3 $\times10^{-3}$ (2.80–17.8)$\times10^{-3}$		1.64 $\times10^{-3}$ (<LOD–14.3)$\times10^{-3}$	2.55 $\times10^{-3}$ (<LOD–14.3) $\times10^{-3}$	0.563 $\times10^{-3}$ (<LOD–0.892)$\times10^{-3}$		0.603$\times10^{-3}$ (<LOD–1.14$\times10^{-3}$)	1.08$\times10^{-3}$ (0.795–1.38$\times10^{-3}$)
**ΣOHCs[e]**	μ ± SD	96.6 ± 65.0	103 ± 56.2	112 ± 111	41.6	37.3 ± 44.6	46.3 ± 50.2	8.11± 47.9	13.9	14.4 ± 8.40	24.0 ± 21.2
	Geometric mean	78.6	91.0	80.6		23.5	30.7	7.17		13.2	18.8
	Median (range)	90.1 (34.1–191)	93.9 (43.6–178)	112 (34.1–191)		20.5 (4.31–202)	24.5 (9.10–202)	8.11 (4.31–11.9)		14.4 (8.51–20.4)	24.0 (9.02–38.9)

[a] Sum of PCB-28, -66, -74, -87, -99, -101, -105, -110, -114, -118, -128, -137, -138, -141, -149, -151, -153, -156-, 157, 170, -180, -183, -187, -189, 194, -196, -206, -209. [b] Sum of p,p`-DDT, p,p`-DDD and p,p`-DDE. [c] Sum of oxychlordane, *trans*-chlordane, *cis*-chlordane, *trans*-nonachlor and *cis*-nonachlor. [d] Sum of BDE-28, -47, -99, -100, -153, -154 [e] Sum of all organohalogen contaminants detected in more than 65% of whale samples. [f] UNK = Unknown sex or age. [g] SA = Subadult. LOD = Limit of detection

Supplementary Table S4: Summary of total mercury levels in all sampled killer whales (n = 38).

**Total mercury levels**		**Seal-eaters**				**Fish-eaters**					
		**All** (n = 10)	**Adult male** (n = 5)	**Adult female** (n = 4)	**UNK**[a] (n = 1)	**All** (n = 28)	**Adult male** (n = 20)	**Adult female** (n = 3)	**SA**[b] **male** (n = 1)	**SA**[b] **UNK** (n = 2)	**UNK** (n = 2)
**Measured in skin (µg/g d.w.)**	µ ± SD	3.73 ± 1.26	3.52 ± 1.66	3.97 ± 0.95		1.76 ± 0.32	1.78 ± 0.27	3.19 ± 1.49		1.53 ± 0.21	2.08 ± 0.45
	Geometric mean	3.47	3.11	3.87	3.79	1.73	1.76	2.98	1.01	1.52	2.06
	Median (range)	3.95 (1.19–5.44)	3.71 (1.19–5.44)	4.18 (2.62–4.88)		1.79 (1.01–2.40)	1.83 (1.29–2.25)	2.62 (2.08–4.88)		1.53 (1.38–1.67)	2.08 (1.76–2.40)
**Estimated in liver (µg/g w.w.)**[c]	µ ± SD	12.5 ± 5.62	11.6 ± 7.1	12.5 ± 4.47		3.57 ± 1.05	3.68 ± 0.987	3.70 ± 0.531		2.50 ± 0.278	4.40 ± 1.48
	Geometric mean	10.5	8.77	11.8	16.8	3.41	3.54	3.68	1.49	2.488	4.27
	Median (range)	14.2 (1.78–18.5)	14.3 (1.78–18.5)	13.2 (6.48–17.1)		3.47 (1.49–5.65)	3.61 (1.57–5.65)	3.411 (3.38–4.31)		2.49 (2.30–2.69)	4.40 (3.34–5.45)

[a] UNK = Unknown sex or age. [b]SA = Subadult. [c] Estimated using equation $\ln(Hg_{liver}) = 1.6124 \times \ln(Hg_{skin}) + 2.0346$ based on bottlenose dolphins (*Tursiops truncatus*)[58].
